# Systematic revision of *Stegodera* Martens, 1876 (Gastropoda, Stylommatophora, Camaenidae), with description of a new genus

**DOI:** 10.3897/zookeys.1059.68385

**Published:** 2021-09-01

**Authors:** Zhe-Yu Chen, Zhi-Tong Lyu, Min Wu

**Affiliations:** 1 College of food science and engineering, Wuhan Polytechnic University, Wuhan 430023, China Nanjing University Nanjing China; 2 School of Life Sciences, Nanjing University, Nanjing 210023, China Wuhan Polytechnic University Wuhan China; 3 The Museum of Biology, School of Life Sciences, Sun Yat-sen University, Guangzhou 510275, China Sun Yat-sen University Guangzhou China

**Keywords:** 16S rRNA, Bradybaeninae, Camaeninae, China, *CO1*, new genus, new species

## Abstract

The monotypic genus *Stegodera* Martens, 1876 is systematically revised based on anatomical and morphological examination of freshly collected specimens. A new species from southern Hunan, which resembles *Stegoderaangusticollis*, is confirmed to represent a new genus evidenced by comparative shell morphology and anatomy as well as by molecular phylogenetic analyses. The new genus might be more closely related to *Stegodera* and *Nesiohelix* Kuroda & Emura, but differs anatomically from the latter two genera by the absence of a dart apparatus.

## Introduction

*Stegodera* Martens, 1876 is a monotypic camaenid genus endemic to southern Hubei and northern Jiangxi, China (Zilch 1960; Richardson 1983). Since the original description (Martens 1875a), only a few additional specimens of this species have been reported (Heude 1882; Chen and Gao 1987; Qian et al. 2008; Qian and Zhou 2014). The typical shell character states of *Stegodera* are “shell sinistral, disk-shaped, with low spire and open, deep umbilicus; solid, opaque, brown. Inner whorls slowly increasing, regular; latter half of the last whorl distorted, straightened, covering the preceding whorl above. Aperture very oblique, crescentic, toothless; peristome reflexed; throat very much contracted” (Pilsbry 1895), while the anatomy of this genus kept unknown. Recently, we received several fresh specimens of *Stegoderaangusticollis* (Martens, 1875) from southeastern Hubei. In another recent field investigation in southern Hunan, Dr Lu Qiu found two land snail specimens belonging to a species, which is conchologically similar to *Stegoderaangusticollis*, but has been found to differ from it in anatomical characteristics.

## Materials and methods

A piece of foot tissue was cut from the living animal and preserved in 99.7% ethanol for molecular analysis. Then the animal was relaxed by drowning in water before being transferred to 70% ethanol for fixation, which was replaced with ethanol of the same concentration after three days. Photographs of the shell and reproductive system were taken using a Canon camera with Macro lens, and then modified in Adobe Photoshop CC 2018. The shells were measured with digital vernier calipers to the nearest 0.1 mm. Measurement abbreviations:

**S_Dma_**_j_ shell major diameter;

**S_Dmin_** shell minor diameter;

**S_H_** Shell height.

Whorls were counted as described by Kerney and Cameron (1979). Directions used in descriptions: proximal, towards the genital atrium; distal, away from the genital atrium.

### Molecular phylogenetics

Muscle tissue was obtained from eleven species in this study (Table 1), including *Stegoderaangusticollis* and the single paratype of *Pseudostegoderaqiului* gen. et sp. nov. Genomic DNA was extracted by using Tiangen DNA Extraction Kit (for SYS samples. Abbreviations see below) and TIANamp Marine Animals DNA Kit (for HBUMM samples). Two mitochondrial genes, partial 16S ribosomal RNA gene (16S) and partial cytochrome *C* oxidase 1 gene (CO1), were amplified. Primers used for 16S were 16SA/16SB (Palumbi et al. 1991), and for CO1 were LCO1490/HCO2198 (Folmer et al. 1994). PCR amplifications were performed in a 20 μl (for SYS samples) / 50 μl (for HBUMM samples) reaction volume with the cycling conditions of an initial denaturing step at 94° C for 2 min, 35 cycles of denaturing at 94° C for 30 s, annealing at 58° C (for 16S)/ 50° C (for CO1) for 30 s and extending at 72° C for 30 s, and final extending step of 72° C for 10 min. PCR amplicons were inspected on a 1% agarose gel for quality and fragment size, then were purified, and sequenced on an automated sequencer.

**Table 1.** Vouchers and the GenBank accession numbers of the species for phylogenetic study (*, from NCBI).

Species	Subfamily	16S/CO1	Museum voucher	Voucher inf.
*Pseudostegoderaqiului* gen. et sp. nov.	Camaeninae	MW810083/ MW810790	SYS m001017	Paratype of the species; see text
* Stegodera angusticollis *	Camaeninae	MW810079/ MW810793	SYS m001016	See text
* Amphidromus inversus *	Camaeninae	AB112400*/ FJ472655*	/	/
* Camaena cicatricosa *	Camaeninae	KU586474*/ KU061276*	/	/
* Camaena poyuensis *	Camaeninae	KU586468*/ KU061273*	/	/
* Satsuma guandi *	Camaeninae	MW804648/ MW810791	HBUMM08239a1	Guangdong, Shaoguan, coll. Di Yu
		MW804647/ MW810792	HBUMM08239a2	
* Exiligada gregoriana *	Camaeninae	JX393672*/ JX393761*	/	/
* Falspleuroxia overlanderensis *	Camaeninae	KU519178*/ KU519261*	/	/
* Tatemelon musgum *	Camaeninae	KU519194*/ KU519277*	/	/
* Sinumelon vagente *	Camaeninae	KP965282*/ KP965358*	/	/
* Acusta ravida *	Bradybaeninae	MW800197/ MW810782	HBUMM06616a	Sichuan, Jiuzhaigou, coll. Min Wu
* Bradybaena qixiaensis *	Bradybaeninae	MW810081/ MW810783	HBUMM06900–1/HBUMM06900–2	Jiangsu, Nanjing, coll. Min Wu
* Cathaica fasciola *	Bradybaeninae	MW800200/ MW810784	HBUMM06477–1/HBUMM06477–2	Jiangsu, Zhenjiang, coll. Min Wu
* Coccoglypta liui *	Bradybaeninae	MK680922*/ MK680001*	/	/
* Coccoglypta pinchoniana *	Bradybaeninae	MK680923*/ MK680002*	/	/
* Dolicheulota formosensis *	Bradybaeninae	KR338956*/ KR338956*	/	/
* Euhadra dixoni *	Bradybaeninae	AF098711*/ AB916773*	/	/
* Laeocathaica polytyla *	Bradybaeninae	MW810082/ MW810787	HBUMM06726a1	Sichuan, Jiuzhaigou, coll. Min Wu
* Laeocathaica distinguenda *	Bradybaeninae	MW810084/ MW810785	HBUMM06491a1	Gansu, Wenxian, coll. Min Wu
		MW810085/ MW810786	HBUMM06491a2	
* Nesiohelix moreletiana *	Bradybaeninae	MW810080/ MW810788	HBUMM06796	Zhejiang, Hangzhou, coll. Min Wu
* Pseudobuliminus piligerus *	Bradybaeninae	MW800362/ MW810789	HBUMM06527a1	Gansu, Wenxian, Coll. Min Wu
*Cornuaspersum* (out-group)	Helicidae	KU586459*/ KU586502*	/	/

**Figure 1.**
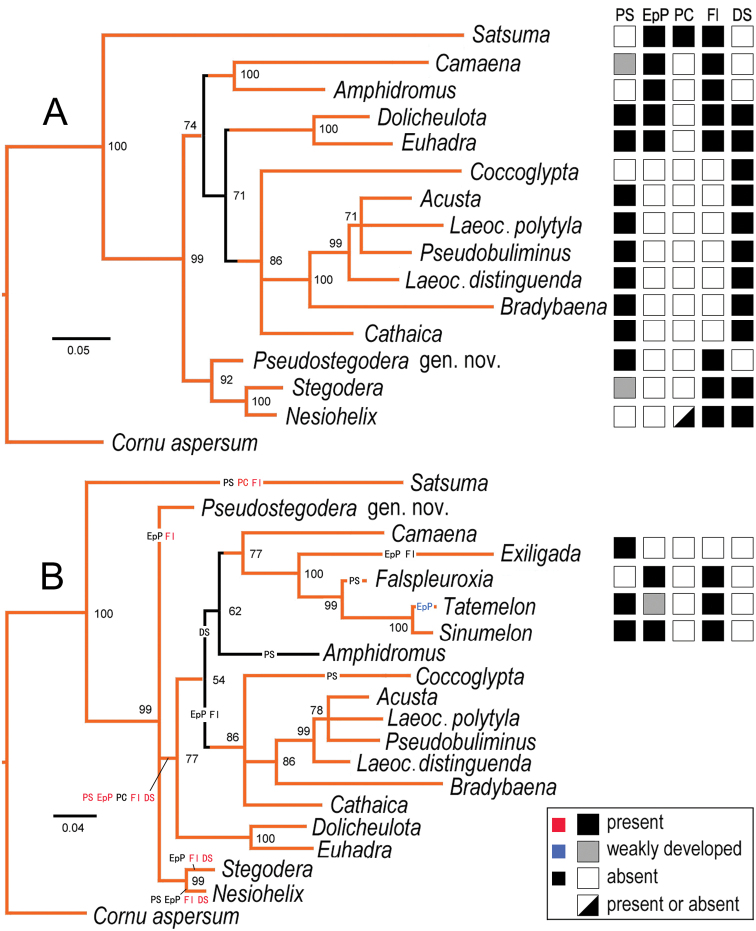
Bayesian Inferences of camaenids (representatives of genera in Table 1) based on partial mitochondrial 16S and CO1 sequences. Numbers near nodes indicate Bayesian posterior probabilities. The squares on the right of each taxon name indicate its character states. Abbreviations: PS: penial sheath; EpP: epiphallic papilla; PC: penial caecum; Fl: flagellum; DS: dart sac apparatus **A** phylogram without Australian camaenids **B** the results of ancestral states (tagged on branch in red, blue, or black) reconstruction mapping on the phylogram with some Australian camaenid genera added. Orange part: the topologies supported by the results respectively by using both Bayesian Inference and Maximum Likelihood methods. Character states are from Schileyko (2003) and Wu (2019). Scale bars for substitutions per site.

For phylogenetic analysis, sequences from seven camaenid species and one out-group species were obtained from GenBank and incorporated into our dataset (Table 1). *Cornuaspersum* is used as the out-group of the studied Camaenidae, following the previous studies (Wang, Xiao et al. 2014; Wang, Yang et al. 2014; Huang et al. 2015) considering that the branch including Helicidae is a close group of Bradybaenidae + Camaenidae (Asian and Australasian) + Polygyridae (Wade et al. 2007; Razkin et al. 2015). DNA sequences of the two genes were aligned respectively by the Clustal W algorithm with default parameters (Thompson et al. 1997) in MEGA 7.0.26 (Kumar et al. 2015). The substitution saturation assessment for CO1 genes was done using DAMBE 7.0 (Xia 2018) employing the methods introduced by Xia et al. (2003) and Xia and Lemey (2009). The poorly aligned positions and divergent regions of the alignment of 16S were eliminated using Gblocks 0.91b (Castresana 2000), a concatenated matrix of 20 (including outgroup, Table 1)×703 bp was used for the subsequent analyses. Model selection was performed by “Models” in MEGA 7.0.26 (Kumar et al. 2015). The data set was analyzed using Bayesian Inference (BI) in MrBayes 3.2.4 (Ronquist et al. 2012) and the Maximum Likelihood analysis in raxmlGUI 2.0 beta (Edler et al. 2019) (Fig. 1). In Bayesian Inference analysis, three independent runs were conducted, each of which was performed for 10,000,000 generations and sampled every 1000 generations with the first 25% samples discarded as burn-in. Convergence of the Markov Chain Monte Carlo simulations was assessed using Tracer v1.7 (Rambaut et al. 2018), verifying that all ESS values exceeded 200. In addition, we repeated these analyses for a dataset containing a larger number of species (Table 1) (Fig. 1B).

We used Mesquite v. 3.61 (Maddison and Maddison 2019) to reconstruct the evolutionary history of five reproductive characters, which are usually emphasized in camaenid taxonomy, and mapped these characters on the Bayesian phylogeny under Maximum Likelihood-based criterion.

### Depositary abbreviations

**HBUMM** Mollusk collection of Museum of Hebei University, Baoding, China;

**IZCAS**Institute of Zoology, Chinese Academy of Sciences (Beijing, China);

**MYNU** Mianyang Normal University (Mianyang, China);

**OYKC** Private collection of Kai-Chen Ouyang (Kunming, China);

**QL** Private collection of Lu Qiu (Luzhou, China);

**SMF**Senckenberg Forschungsinstitut und Naturmuseum (Frankfurt am Main, Germany);

**SYS**Sun Yat-sen University (Guangzhou, China);

**ZMB**Museum für Naturkunde (Berlin, Germany).

### Anatomical abbreviations

**AS** accessory sac;

**At** atrium;

**BC** bursa copulatrix;

**BCD** bursa copulatrix duct;

**DS** dart sac;

**DSC** dart sac chamber;

**Ep** epiphallus;

**Fl** flagellum;

**FO** free oviduct;

**MG** mucous glands;

**MGP** papilla distally leading to mucous glands on inner wall of accessory sac;

**P** penis;

**PC** penial caecum;

**PR** penial retractor muscle;

**PS** penis sheath;

**Va** vagina; V

**VD** vas deferens.

## Results

### Phylogeny

The substitution saturation assessment for CO1 sequences suggested that the first and second codon positions were relatively conserved but that the third codon positions revealed sequence saturation and are therefore not suitable for phylogenetic inference (for both symmetrical and asymmetrical trees, Iss > Iss.c, *p*s < 0.001). Our final molecular dataset contained nineteen sequences of partial 16S and CO1 genes. After eliminating poorly aligned positions and divergent regions of the alignment of 16S using Gblocks 0.91b (Castresana 2000), a concatenated matrix of 20 (including outgroup, Table 1)×703 bp was used for the subsequent analyses. The models “T92 + G” and “GTR + G + I” were chosen as the best nucleotide substitution models for 16S (lnL = -1877.8, BIC = 4100.7) and *CO1* (lnL = -1399.1, BIC = 3216.9), respectively. The model of the combined dataset is “TN93 + G + I” (lnL=-3387.6, BIC = 7192.6).

The phylograms produced by both Maximum Likelihood Inference and Bayesian Inference based on partial 16S + partial CO1 sequences are topologically identical in major branches (Fig. 1). All trees reveal a primary division (posterior probability PP = 1) between penial caecum-bearing *Satsuma* A. Adams, 1868 (Camaeninae) and the remaining studied groups, a mixture of so-called camaenine and bradybaenine genera that usually have no penial caecum. The monophyly of Bradybaeninae is not supported because neither *Nesiohelix* Kuroda & Emura, 1943 nor *Dolicheulota* Pilsbry, 1901, and *Euhadra* Pilsbry, 1890, all well-known bradybaenine genera, are not included in the clade where most bradybaenine genera stay. The monophyly of Camaeninae, which is morphologically characterized by the absence of dart sac apparatus is also not supported when considering that *Satsuma* is situated most basally on the phylograms and meanwhile *Camaena* Albers, 1850 and its sister group *Amphidromus* Albers, 1850 are deeply nested in the clade that comprises most bradybaenine genera. *Stegodera* E. Martens, 1876 is found to be the sister group of *Nesiohelix* with strong nodal support (PP = 1). In one phylogram (Fig. 1A), *Pseudostegodera* gen. nov. is a sister group of clade *Steogera* + *Nesiohelix* and in the other, it is in an unresolved trichotomy with these two taxa.

As suggested by the analyses of character evolution, the ancestral character states among most studied camaenids, except *Satsuma*, *Pseudostegodera* gen. nov., *Stegodera*, and *Nesiohelix*, are: penial sheath, epiphallic papilla, flagellum and dart sac apparatus present, penial caecum absent (Fig. 1B). The penial sheath has been lost in all the studied taxa at least for five times. The epiphallic papilla has been lost at least for four times but regained once in *Tatemelon*. Amongst the ingroup, the dart sac was acquired for three times but lost in *Camaena*, *Amphidromus* and the Australian camaenids. The flagellum has been lost twice, once in *Exiligada* Iredale, 1939, and once in the clade including *Coccoglypta* Pilsbry, 1895, *Acusta* Martens, 1860, *Laeocathaica* Moellendorff, 1899, *Pseudobuliminus* Gredler, 1887, *Bradybaena* Beck, 1837, and *Cathaica* Moellendorff, 1884, which are all bradybaenine genera mainly distributed in the mainland of China.

### Systematics

#### Family Camaenidae Pilsbry, 1895

##### Stegodera

Taxon classificationAnimaliaStylommatophoraCamaenidae

Martens, 1876

AA63B94A-F093-547F-99D6-3E116AAE19D2

Helix (Stegodera) Martens in Pfeiffer 1876: 150; Schmacker and Boettger 1894: 173.
Stegodera . – Pilsbry 1905: 64, Yen 1939: 126; Zilch 1960: 610; Schileyko 2003: 1512.
Steganodera Kobelt, 1879: 236 (incorrect subsequent spelling or unjustified emendation); Schileyko 2003: 1512 (syn pro Stegodera Martens, 1876).
Plectopylis (Stegodera) . – Pilsbry 1894: 147.
Planispira (Stegodera) . – Thiele 1931: 681.

###### Type species.

*Helixangusticollis* Martens, 1875, by original designation.

###### Diagnosis.

Shell sinistral. Apical whorls with dense fine ribs that gradually becoming granules. The last ^1^/_4_ body whorl compressed, apically covering the contacted penultimate whorl. Peristome expanded and slightly reflexed. Head wart absent. Each side of mantle edge with a leaf-shaped appendage. Penis sheath weakly present. Penis externally simple. Epiphallic papilla absent. Flagellum present. Dart sac apparatus present. Accessory sac well developed. Mucous glands with numerous gland tubes. Membranous sac surrounding terminal genitalia absent. Poly-layered structure in dart sac and/or accessory sac absent.

###### Remarks.

This genus was considered as a subgenus of *Helix* Linnaeus, 1758 or *Plectopylis* Benson, 1860 for some time (Martens 1876; Schmacker and Boettger 1894; Pilsbry 1894). Pilsbry (1905) formally established its independent status. *Stegodera* and *Traumatophora* Ancey, 1887 were considered to be closely related, and *Traumatophora* was once treated as a subgenus of *Stegodera* (Pilsbry 1905; Gude 1920). Pilsbry (1905) believed that the two shallow grooves in the throat area of *Stegodera* are likely to be homologous to the dentition in *Traumatophora*.

In light of the genital system, *Stegodera* shares with *Nesiohelix* the most important character states like the presences of flagellum, numerous tubes of mucous glands and the papilla distally leading to mucous glands on the inner wall of the accessory sac, and the absences of the epiphallic papilla, poly-layered structure in dart sac apparatus and membranous sac surrounding terminal genitalia (Wu 2019). The close relationship between them in genitalia is supported by the phylogenetic result of this work (Fig. 1A, B).

##### Stegodera angusticollis

Taxon classificationAnimaliaStylommatophoraCamaenidae

(Martens, 1875)

B0722042-8149-51EB-A94D-D218446E36F0

**Figure 2.**
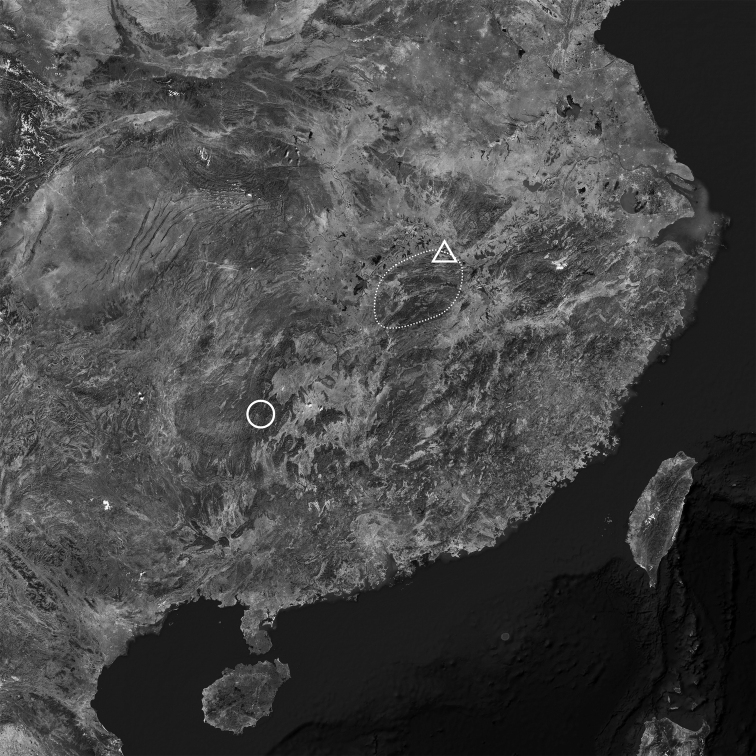
Distribution of *Stegodera* and *Pseudostegoderaqiului* gen. et sp. nov. Triangle: new locality of *Stegoderaangusticollis* (Martens, 1875); dashed range: area of *Stegoderaangusticollis*; circle: type locality of *Pseudostegoderaqiului* gen. et sp. nov.

**Figure 3.**
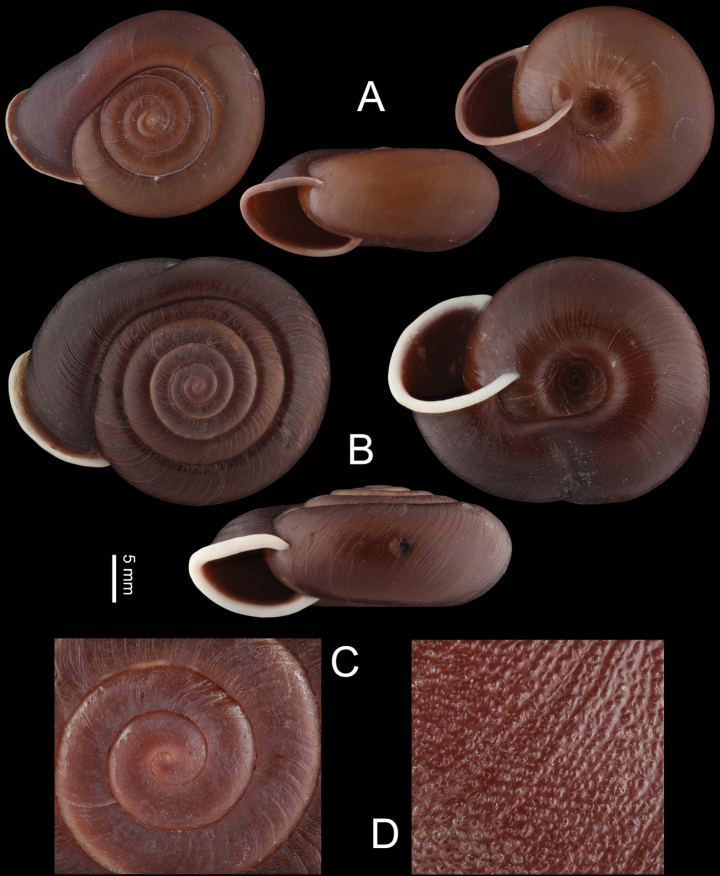
Shells **A***Stegoderaangusticollis* Martens, 1876, HBUMM08435 **B–D***Pseudostegoderaqiului* gen. et sp. nov., holotype IZCAS TM206978 **C** p rotoconch, magnified **D** shell surface, magnified. Scale bar: 5 mm (**A, B**).

**Figure 4.**
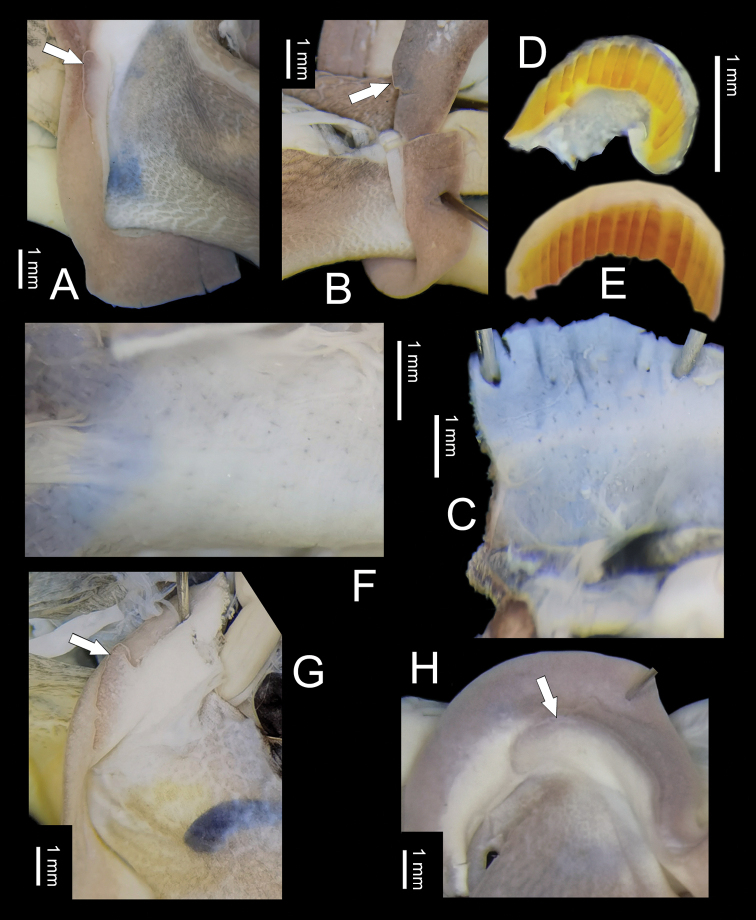
General anatomy **A–D***Stegoderaangusticollis* (Martens, 1875), HBUMM08435 **A** lobe (arrowed) on the left side of mantle edge **B** lobe (arrowed) on the right side of mantle edge **C** internal surface of head region **D** jaw **E–H***Pseudostegoderaqiului* gen. et sp. nov., holotype IZCAS TM206978 **E** jaw **F** internal surface of head region **G** lobe (arrowed) on the left side of mantle edge **H** lobe (arrowed) on the right side of mantle edge.

**Figure 5.**
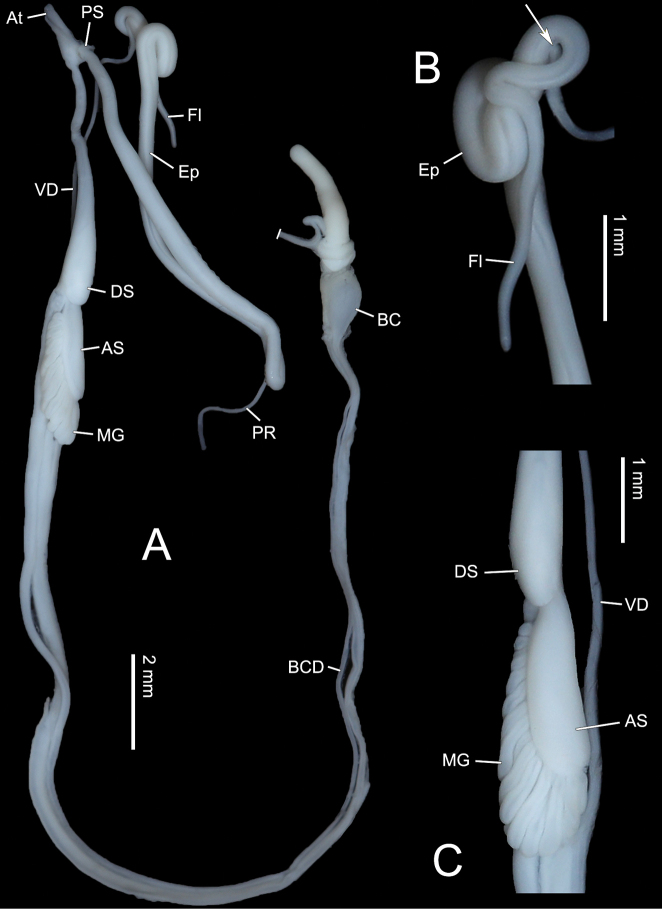
Genitalia of *Stegoderaangusticollis* (Martens, 1875), HBUMM08435 **A** general view **B** male part, showing the vas deferens connecting epiphallus (arrowed) **C** female part, showing the dart sac, accessory sac, and mucous glands.

**Figure 6.**
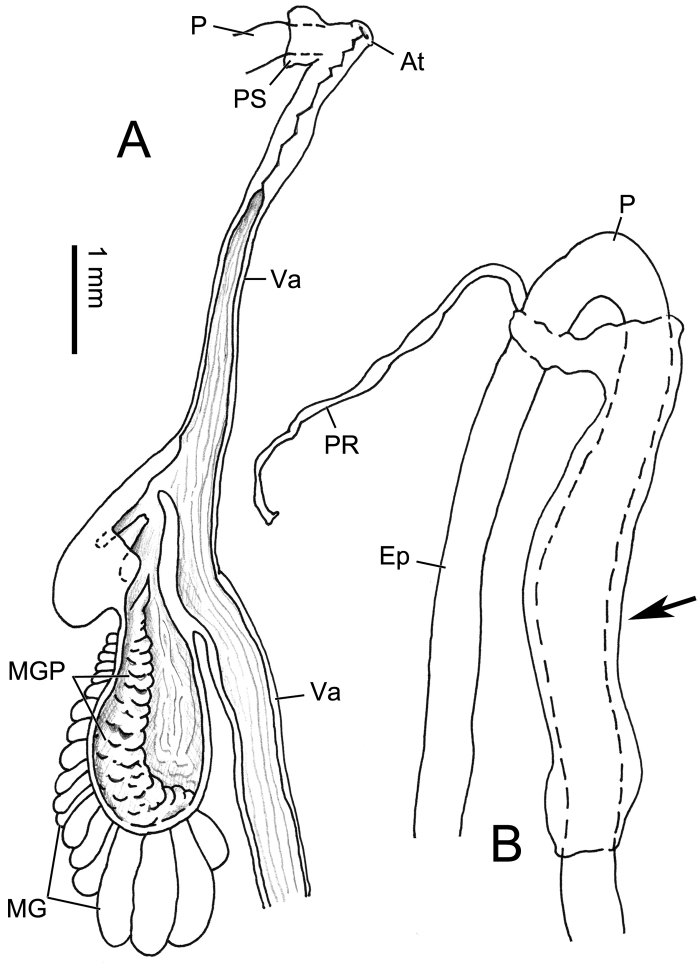
Genitalia of *Stegoderaangusticollis* (Martens, 1875), HBUMM08435 **A** exposed dart sac apparatus **B** magnified partial penis and partial epiphallus. Arrow showing a very thin membranous sac wrapping distal half of penis with distal end connecting basal penial retractor muscle.

Figures 1 , 2 , 3A , 4A–D , 5A–C , 6A, B , 8A–C

Helix angusticollis Martens, 1875a: 2. Martens 1875b: 185; Pfeiffer 1875: 449, 1876: 149, pl. 134, figs 7–10; Gredler 1882: 175; Heude 1882: 36, pl. 15, fig. 8; Möllendorff 1884: 387.
Helix (Stegodera) angusticollis . – Martens, 1876 (in Pfeiffer 1870–1876): 149–150; Pilsbry 1890: 7, pl. 1, figs 15–17.
Plectopylis (Stegodera) angusticollis . – Pilsbry 1894: 147.
Planispira (Stegodera) angusticollis . – Thiele 1931: 681.
Stegodera angusticollis . – Gude 1902: 4; Kobelt 1905: 78; Pilsbry 1905: 64, 66, pl. 2, figs 1–3; Gude 1920: 60; Yen 1939: 126, pl. 13, fig. 9; Zilch 1960: fig. 2138; Richardson 1983: 291; Schileyko 2003: 1512, fig. 1948; Chen and Gao 1987: 107, fig. 137; Qian et al. 2008: 289, fig. 169; Qian and Zhou 2014: 120, figure in text.

###### Museum material examined.

•ZMB. Moll. 31044, syntype, Poyangsee, China, slg. v. Richthofen; ZMB. •ZMB. Moll. 3710/1 China, slg. Paetel; •SMF27113/3, China: Prov. Hupei, slg. O. v. Moellendorff; •SMF27114/2, China: Wǔ-chang-fú, slg. K. Hashagen (ex. Schmacker); •SMF42579/3, China: Húbei (Hupe), slg. Ehrmann; •IZCAS TM108733/1, Kieou-Kiang; •IZCAS TM158903-158920/18, Ou-tchang h[ien], ex. Musée Heude; •IZCAS TM159272-159397/126, Kieou-Kiang, ex. Musée Heude.

###### New material examined.

•One shell of HBUMM08435 (dissected), Guanyin Cave [观音洞], Taizi Town [太子镇], Yangxin County [阳新县], Huangshi City [黄石市], Hubei Province, China, 30°0'3.816"N, 115°11'24.428"E, 140 m a.s.l., 2020-X-15, leg. Xiao-Long Wang & Zi-Hao Shen (S_Dmaj_ = 26.7 mm, S_Dmin_ = 21.3 mm, S_H_ = 11.3 mm). •Three shells of OYKC, one shell of QL, Lushui Lake [陆水湖], Chibi City [赤壁市], Xianning City [咸宁市], Hubei Province, China, 29°40'12"N, 113°58'35"E, 2020-X-03, leg. Di Yu & Kai-Chen Ouyang.

###### Type locality.

Poyang-See (= Poyang Lake [鄱阳湖], Jiangxi).

###### Measurements of new material.

S_Dmaj_ = 26.7–30.6 mm, S_Dmin_ = 21.3–23.7 mm, S_H_ = 11.3–12.2 mm (n = 6).

###### Diagnosis.

Body whorl completely covering partial penultimate whorl. Penial sheath weakly present. Epiphallic papilla absent. Dart sac apparatus and flagellum present. Mucous glands with numerous tubes.

###### Redescription.

Shell (Fig. 3A). Sinistral, large, solid, rather flat, five whorls, in chestnut and darker near aperture. Suture impressed. Protoconch 1^1^/_4_ whorls. After ~ 3^1^/_2_ whorls, growth lines broken into regularly arranged tubercles. Body whorl compressed from the last ^1^/_4_ to the last ^1^/_8_ whorls, completely covering corresponding part of penultimate whorl including the suture. After the last ^1^/_8_ whorls, body whorl becoming as broad as normal again. On the above compressed region, one shorter ventral depression and one longer apical depression present. Aperture semilunar, slightly descending. Peristome chestnut, thickened, expanded, and slightly reflexed. Umbilicus moderately broad; approximately ^1^/_5_ of shell major diameter. Protoconch visible through umbilicus.

General anatomy (Fig. 4A–D). Head wart absent. On internal body wall of head region between ommatophore insertions with tiny pits rather than glands (Fig. 4C). Each side of mantle edge with a leaf-shaped appendage (Fig. 4A, B). Body reddish brown, central dorsa with light longitudinal stripes. Sole dirty white. Jaw arcuate; with 12 more or less projecting ribs (Fig. 4D).

Genitalia (Figs 5A–C, 6A, B). Penis sheath present but thin and very short. Penis medially slightly thickened, moderately long, ~ 8.8 mm, externally simple. Inside penis with several thin longitudinal pilasters. Epiphallus slightly longer than penis. A very thin membranous sac wrapping distal half of penis with distal end connecting basal penial retractor muscle (Fig. 6B). Flagellum cylindrical, tapering. Vas deferens thin throughout. Dart sac apparatus distally inserting on vagina. Dart sac small. Dart not observed. Membranous sac surrounding terminal genitalia absent. Accessory sac relatively large. Tubes of mucous glands more than twenty, neatly inserting in a single row on distal and same side of dart sac on accessory sac (Figs 5C, 6B). From proximal to distal accessory sac, mucous gland tubes increased gradually in length (Figs 5C, 6B). Papilla distally leading to mucous glands on inner wall of accessory sac integrated into one long thick spongious pilaster on the side of mucous gland tube insertion (Fig. 6A). Bursa copulatrix ball-shaped.

###### Ecology.

This species was observed under litter layer, on nearby rocks and crevices.

###### Distribution.

Central China: Hubei, Jiangxi (Fig. 2).

###### Remarks.

The peristome of *Stegoderaangusticollis* is in chestnut in fresh specimens, but in most museum specimens this color gets faint to white. In *Stegoderaangusticollis*, the compressed part of body whorl usually completely cover the penultimate whorl, while in *Pseudostegoderaqiului* gen. et sp. nov. the corresponding part is not so compressed and the compressed part of body whorl can hardly cover the penultimate whorl.

Some authors (Chen and Gao 1987; Qian et al. 2008; Qian and Zhou 2014) recorded this species in Jiangsu, Zhejiang and Anhui, but voucher specimens from these provinces were neither recorded therein nor being found in the mollusk collection deposited in IZCAS where some of them once worked. Therefore, the accuracy of these records needs to be further confirmed.

##### Pseudostegodera

Taxon classificationAnimaliaStylommatophoraCamaenidae

Wu & Chen gen. nov.

EFBB53B7-D144-58F5-B1ED-5B0A2A965DB5

http://zoobank.org/F0A6D222-8DB2-4B57-8910-17CAAE2B7FFA

###### Type species.

*Pseudostegoderaqiului* gen. et sp. nov.

###### Diagnosis.

Shell sinistral. Apical whorls with dense fine ribs gradually becoming granules. The last ^1^/_8_ to ^1^/_4_ body whorl compressed, only partly covering penultimate whorl. Umbilicus broad. Head wart absent. Each side of mantle edge with a leaf-shaped appendage. Penis sheath present. Penis externally simple. Epiphallic papilla absent. Flagellum present. Dart sac apparatus absent.

###### Remarks.

Conchologically the new genus can be distinguished from *Stegodera* by the detached body whorl and penultimate whorl. The new genus is supported to be the closest relative of a clade containing *Stegodera* + *Nesiohelix* as based on the Maximum Likelihood/Baysian Inference phylogeny shown in Fig. 1A. However, when the Australian camaenids were included in the analysis, the relationships between the new genus, *Stegodera* + *Nesiohelix*, and all remaining taxa except *Satsuma* remained unresolved (Fig. 1B). The new genus is conchologically similar to *Stegodera*, however, it differs in certain reproductive characters (see above). We consider that the genital anatomy is more informative than shell features in helicoid systematics (e.g., Criscione and Köhler 2013). In addition, the loss of the dart sac apparatus in Bradybaeninae and/or Camaeninae is not a frequent event during the evolution of Camaenidae (Fig. 1B). Based on these two considerations, we consider it is reasonable to distinguish this new camaenid taxon as a monotypic genus represented by the new species described below.

##### Pseudostegodera qiului

Taxon classificationAnimaliaStylommatophoraCamaenidae

Chen sp. nov.

**Figure 7.**
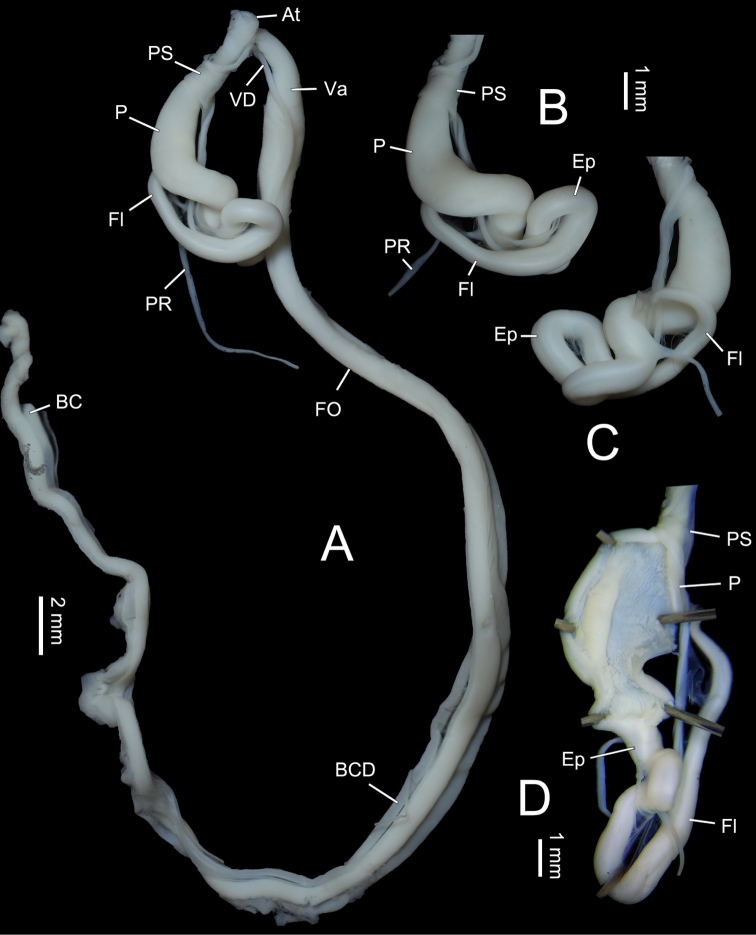
Genitalia of *Pseudostegoderaqiului* gen. et sp. nov., holotype IZCAS TM206978 **A** general view **B, C** male part, two sides **D** exposed penis.

D2335B4D-DC0B-5260-8421-1BBEB4270004

http://zoobank.org/E95104CE-D502-4E48-A256-F0727253745C

(Figs 1 , 2 , 3B–D , 4E–H , 7A–D , 9A–C)

###### Type material.

•***Holotype***, IZCAS TM206978, Zihuaping [紫花坪], core area of Shunhuang Mountain National Nature Reserve [舜皇山国家级自然保护区核心区], Huanglong Town [黄龙镇], Xinning County [新宁县], Shaoyang City [邵阳市], Hunan, China, 26°23'31"N, 110°0'25"E, 945 m a.s.l., 2020-VIII-23, leg. Lu Qiu. •Paratype, MYNU/1, same data as holotype.

###### Measurements.

S_Dmaj_ = 31.0 – 34.0 mm, S_Dmin_ = 25.0 – 26.4 mm, S_H_ = 12.0 – 13.7 mm (n = 2).

###### Diagnosis.

Body whorl incompletely covering partial penultimate whorl. Dart sac apparatus absent. Flagellum present.

###### Description.

Shell (Fig. 3B–D). Sinistral, large, depressed, thick and solid, dark reddish-brown. Shell with 5^1^/_2_ convex whorls. Suture impressed. Protoconch 1^1^/_4_–1^1^/_2_ whorls, with regularly arranged fine axial striae that may be invisible by weathering or erosion. Growth lines clear, broken into microscopic tubercles of irregular shape. The last ^1^/_8_ to ^1^/_4_ body whorl compressed, partly covering penultimate whorl. At ^1^/_4_ whorl from the aperture, a spiral depression above periphery and a weak depression near umbilicus making a narrowing on body whorl. Whorl after the narrowing reverting to normal broadness. Aperture semilunar, slightly descending. Peristome white, strongly thickened, expanded, and slightly reflexed. Umbilicus broad, approximately ^1^/_3_ of shell major diameter. Protoconch visible through umbilicus.

General anatomy (Fig. 4E–H). Eversible head wart absent. On internal body wall of head region between ommatophorous insertions with tiny pits rather than glands (Fig. 4F). Each side of mantle edge with a leaf-shaped appendage (Fig. 4G, H). Body reddish brown, central dorsa with light longitudinal stripes. Sole dirty white. Jaw arcuate, with ~ 14 more or less projecting ribs (Fig. 4E).

Genitalia (Fig. 7A–D). Penis sheath short but well developed. Penis somewhat swollen on proximal half, externally simple. In the middle of penis, internally with a single strong longitudinal pilaster almost as thick as epiphallus, ~ 2 mm long (Fig. 7D). Epiphallic papilla absent (Fig. 7D). Flagellum cylindrical, tapering. Vas deferens thin throughout, approximately as long as penis. Dart sac apparatus absent. Bursa copulatrix duct thickened basally. Bursa copulatrix duct longer than 50 mm. Bursa copulatrix rod-shaped.

###### Etymology.

This new species is named after Dr Lu Qiu [邱鹭], who provided the specimens and field data.

**Figure 8.**
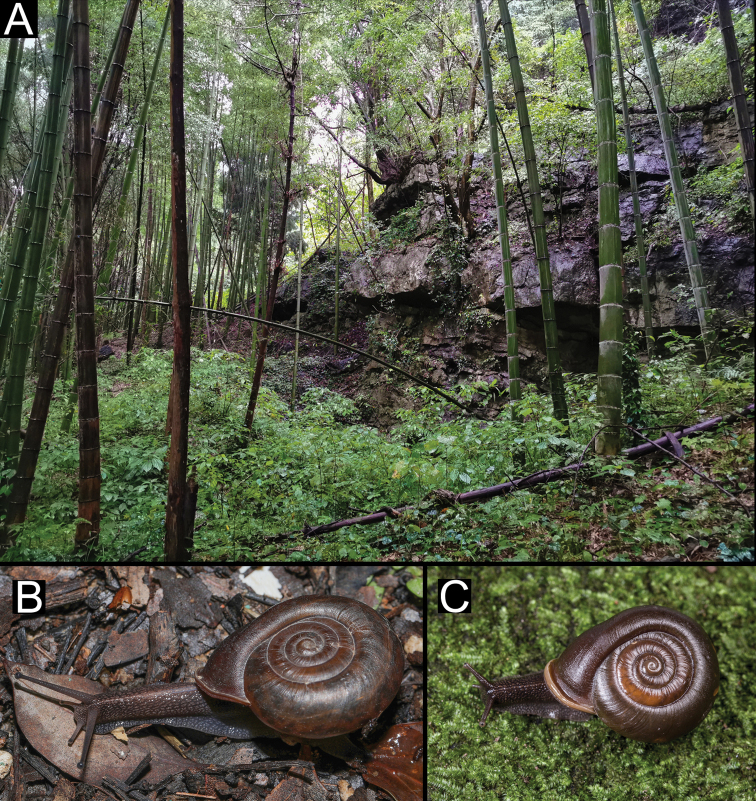
Habitat and living specimens of *Stegoderaangusticollis* (Martens, 1875) **A** habitat (photograph Kai-Chen Ouyang) **B** the living specimen of HBUMM08435 **C** a living specimen from Chibi City (photograph Lu Qiu).

###### Ecology.

This species was found under rotten wood.

**Figure 9.**
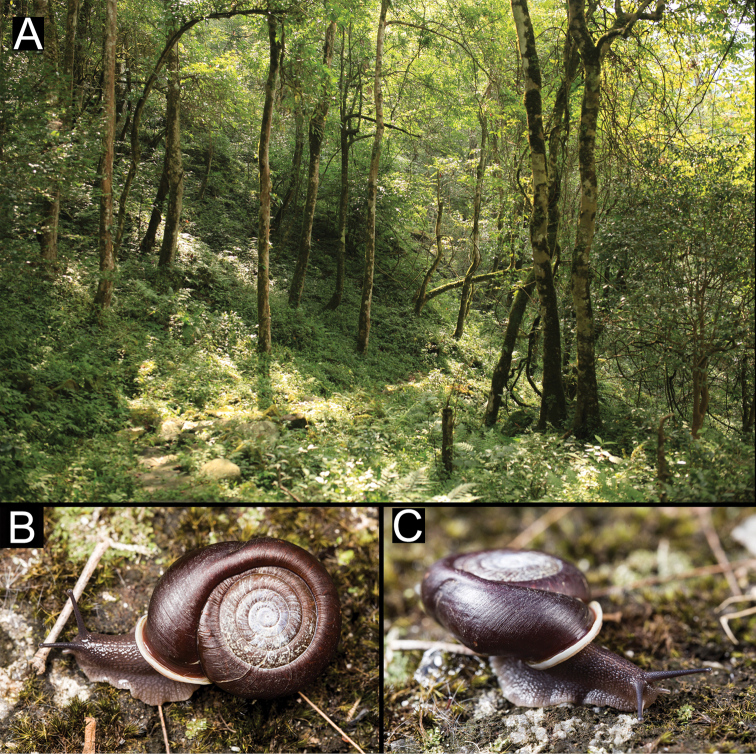
Habitat and living specimens of *Pseudostegoderaqiului* gen. et sp. nov. **A** habitat **B, C** a living specimen. Photographs Lu Qiu.

###### Distribution.

Only known from the type locality (Fig. 2).

###### Remarks.

See under the genus.

## Discussion

The comparison of the phylograms (Fig. 1) suggests the phylogeny obtained in this paper is more or less robust when the Australian camaenids, which form a monophyly together with the Asian camaenids (as suggested by Scott 1996), are considered. The phylogenetic position of *Satsuma* agrees with that suggested by many authors (Chiba 1999: fig. 3; Wade et al. 2007: fig. 2). As indicated by some authors, *Satsuma* is the sister group of the common ancestor of Bradybaeninae and Camaeninae (Fig. 1; Wade et al. 2007: fig. 2), rather than a typical camaenine (sensu Azuma 1995) or bradybaenine (sensu Schileyko 2004). The monophyly made by *Stegodera*, *Nesiohelix* and the new genus is the sister group of all the remaining bradybaenines and camaenines involved in this study. It suggests that neither the Camaeninae including *Satsuma* (sensu Azuma 1995) nor the Bradybaeninae including *Nesiohelix* (sensu nearly all the authors) are monophyletic.

The current work also corfirms that so-called important characters of genital system, including penial sheath, epiphallic papilla, penial caecum, flagellum, and dart sac apparatus, have homoplasiously evolved more than once in the studied terminal taxa and their ancestral nodes (Fig. 1B). In other words, widespread homoplasious changes in morphology (Hirano et al. 2014; Fig. 1) including dart sac apparatus based on which Bradybaeninae and Camaeninae are distinguished, rendering the basis of the establishment of Bradybaeninae particularly feeble, explaining that no agreement on the subdivision within Camaenidae was reached to date (Nordsieck 2002; Schileyko 2003, 2004; Wade et al. 2006, 2007; Gittenberger et al. 2012; Bouchet et al. 2017).

## Supplementary Material

XML Treatment for Stegodera

XML Treatment for Stegodera angusticollis

XML Treatment for Pseudostegodera

XML Treatment for Pseudostegodera qiului
